# Audit of Appointment Non-Attendance in Leeds Child and Young Person Learning Disability Psychiatry Clinic

**DOI:** 10.1192/bjo.2025.10595

**Published:** 2025-06-20

**Authors:** Amy Greener, Martha Sayer, Eleanor Freud, Konstantopoulou Kalliopi

**Affiliations:** 1 University of Leeds, Leeds, United Kingdom; 2 Leeds & York Partnership NHS Foundation Trust, Leeds, United Kingdom; 3 Leeds Community Healthcare, Leeds, United Kingdom

## Abstract

**Aims:** Appointment non-attendance has significant impacts on care and resource allocation across the NHS. This audit investigates appointment non-attendance within the Leeds Child and Young Person Mental Health Service (CYPMHS) Learning Disability (LD) Psychiatry Clinic, aiming to identify contributing factors and propose strategies to improve attendance in the context of the unique challenges and complexities associated with LD.

**Methods:** This retrospective clinical audit analyses data on 237 scheduled appointments at the Leeds CYPMHS LD Psychiatry Clinic between September 2023 and August 2024. Data collection involved reviewing electronic medical records to identify trends within anonymised patient information, handled securely in compliance with NHS and University of Leeds data protection policies.

MS and AG investigated multiple strata to identify trends explaining DNAs, including: attendance status (parent and child) by month and overall, age, gender, ethnicity, location, appointment time, day of the week, and format (virtual vs in-person). More detailed analyses explored potential patterns by clinician, school, postcode-linked deprivation indices, diagnosis, medication, and recorded reasons for non-attendance.

**Results:** 83% of overall appointments were attended.

Afternoon appointments had a higher attendance rate (94%) compared with morning slots (81%). Older teenagers were more likely to attend, with a trend of increasing attendance by year of age: 91% in 17-year-olds vs. 67% in 9-year-olds. Home visits had the highest attendance (100%), followed by school visits (85%), virtual consultations (81%) and clinic-based appointments (80%).

Attendance patterns differed between children and parents. While home visits had the highest attendance for both (100%), school visits were preferable for children (86%) whereas parents attended best virtually (83%).

**Conclusion:** This audit highlights predictors of appointment attendance. Findings suggest that targeted adjustments may enhance engagement and reduce DNAs, such as: prioritising home and school-based visits, afternoon appointment timing, and developing strategies to improve access amongst younger children. These findings align with existing literature, showing higher non-attendance rates among younger children and for morning appointments in paediatric and learning disability (LD) services.

The inverse attendance patterns between parents and children by location raises questions about whether services should prioritise child or parental attendance in decision-making. Joint attendance is often important for LD assessments, so this may require further discussion amongst the clinical team.

Further research is needed to identify barriers to attendance for younger children, and to explore whether alternative scheduling/alternative interventions could improve engagement and reduce DNAs. These insights can inform broader service delivery strategies to improve care access and efficiency.

