# Audit of Appointment Non-Attendance in Leeds Child and Young Person Learning Disability Psychiatry Clinic – CORRIGENDUM

**DOI:** 10.1192/bjo.2025.10889

**Published:** 2025-10-10

**Authors:** Amy Greener, Martha Sayer, Eleanor Morris, Konstantopoulou Kalliopi

In the original publication of this abstract, co-author Eleanor Morris’ name was incorrectly given as ‘Eleanor Freud’.

The abstract has been updated to correct this, and this corrigendum published.
